# LncRNA UCA1 in anti-cancer drug resistance

**DOI:** 10.18632/oncotarget.18344

**Published:** 2017-06-02

**Authors:** Haohao Wang, Zhonghai Guan, Kuifeng He, Jiong Qian, Jiang Cao, Lisong Teng

**Affiliations:** ^1^ Cancer Center, The First Affiliated Hospital, College of Medicine, Zhejiang University, Hangzhou, Zhejiang, P.R. China; ^2^ Key Laboratory of Precision Diagnosis and Treatment for Hepatobiliary and Pancreatic Tumor of Zhejiang Province, Hangzhou, Zhejiang, P.R. China; ^3^ Clinical Research Center, The Second Affiliated Hospital, College of Medicine, Zhejiang University, Hangzhou, Zhejiang, P.R. China

**Keywords:** long non-coding RNA, UCA1, chemoresistance, cancer

## Abstract

The pivotal role of the long non-coding RNA (lncRNA) urothelial carcinoma associated 1 (UCA1) in anti-cancer drug resistance has been confirmed in many cancers. Overexpression of lncRNA UCA1 correlates with resistance to chemotherapeutics such as cisplatin, gemcitabine, 5-FU, tamoxifen, imatinib and EGFR-TKIs, whereas lncRNA UCA1 knockdown restores drug sensitivity. These studies highlight the potential of lncRNA UCA1 as a diagnostic and prognostic biomarker, and a therapeutic target in malignant tumors. In this review, we address the role of lncRNA UCA1 in anti-cancer drug resistance and discuss its potential in future clinical applications.

## INTRODUCTION

A recent comprehensive analysis of RNA sequencing data from human tissues identified nearly 60,000 long non-coding RNAs (lncRNAs), 3 times the number of protein-coding mRNAs [1]. LncRNAs represent a large family of evolutionarily conserved non-coding RNAs, about 200 nucleotides in length [2]. Recently, some studies demonstrated that some lncRNAs may encode small peptides, probably because minimum cutoff previously used for a classic open reading frame prediction was 100 codons [3–6]. LncRNAs play crucial roles in diverse biological pathways and cellular processes including embryonic development, cell growth and carcinogenesis by affecting gene expression at the transcriptional, post-transcriptional and translational levels [7]. LncRNAs function in either nuclei and/or cytoplasm. In the nucleus, lncRNAs are involved in chromatin remodeling and modification, transcriptional modulation, or RNA processing; in the cytoplasm, lncRNAs interact with mature RNAs and/or proteins [8]. In the last decade, many studies have shown that lncRNAs play important roles as oncogenes or tumor suppressors [9]. Several lncRNAs such as HOTAIR, MALAT1, GAS5, PC3, and H19 are aberrantly regulated in various malignant tumors and associated with carcinogenesis, metastasis, and prognosis [10–14]. The Food and Drug Administration (FDA) approved urine biomarker lncRNA PCA3 is a more specific and sensitive biomarker for prostate cancer compared to serum prostate-specific antigen [13, 15]. Also, phase I/II clinical trials are underway in patients with bladder, pancreatic and ovarian cancers for lncRNA H19 targeted therapy based on *in vitro* experiments demonstrating tumor cell growth arrest without affecting normal cells with H19 promoter cloned into BC-819 plasmid [16–18]. Hence, lncRNAs have shown immense potential as diagnostic and prognostic biomarkers and therapeutic targets in malignant tumors.

The lncRNA UCA1 was first cloned and identified from bladder cancer cell line BLZ-211 [19]. It is located on human chromosome 19p13.12 positive strand and has three exons and two introns with multiple stop codons without any conserved long open reading frames (ORFs) [19]. The three transcriptional isoforms of UCA1 are 1.4 kb, 2.2 kb and 2.7 kb long, generated by splicing and polyadenylated. The 1.4 kb isoform is denoted as lncRNA UCA1, while the 2.2 kb isoform is denoted lncRNA UCA1a or lncRNA CUDR; the biological role of the 2.7 kb isoform is not known [20]. LncRNA UCA1 is the most abundant isoform in various malignant tumors such as bladder cancer, breast cancer and hepatocellular carcinoma [19, 21, 22, 23]. Hence, most studies have mainly focused on the expression, regulation and functional significance of lncRNA UCA1.

Ectopic expression of lncRNA UCA1 in bladder cancer cell line BLS-211 promoted cancer progression demonstrating that lncRNA UCA1 was oncogenic [21]. Several recent studies have also identified oncogenic functions of lncRNA UCA1 in various cancers such as bladder cancer, breast cancer, colorectal cancer, esophageal squamous cell carcinoma, gastric cancer, hepatocellular carcinoma, melanoma, ovarian cancer, and tongue squamous cell carcinoma [19–29]. Besides the oncogenic function, lncRNA UCA1 also regulates drug resistance in multiple types of malignant tumors. In this article, we review the pivotal roles of the lncRNA UCA1 in drug resistance and discuss the potential future clinical applications.

### LncRNA UCA1 expression in various cancers

LncRNA UCA1 is upregulated at 5–10 weeks of gestation; after 28 weeks of gestation, it is highly expressed in bladder, heart and uterus compared to cervix, kidney, liver, lung, intestine, skin, spleen and stomach; after birth, it is turned off in most tissues except heart and spleen [19, 21]. Moreover, lncRNA UCA1 is reactivated in various malignant tumors including bladder cancer, breast cancer, cervical cancer, colorectal cancer, esophageal squamous cell carcinoma, hepatocellular carcinoma, gastric cancer, lung cancer, melanoma, ovarian cancer, thyroid cancer, tongue squamous cell carcinoma, prostate cancer, acute myeloid leukemia, pancreatic cancer, glioma and osteosarcoma [19, 21–33].

The subcellular localization data indicates that lncRNA UCA1 is mainly located in cytoplasm suggesting that it interacts with mature RNAs and/or proteins and regulates them [34]. Interestingly, lncRNA UCA1 is also detected in blood and urine samples from bladder cancer patients and is a useful circulating biomarker [35]. Its detection in urine samples is an effective and noninvasive mode of diagnosing bladder cancer with high sensitivity (100%) and specificity (67%) in combination with the standard cytology method [36]. However, the underlying mechanisms regarding its release from cancer cells into circulation remain to be elucidated. Extracellular vesicles such as exosomes and microvesicles carry coding and non-coding RNAs, proteins and lipids through plasma membranes from various cell types [37]. A recent *in vitro* study reported that incubation of exosomes with lncRNA UCA1 from the tamoxifen-resistant breast cancer cell line LCC2 with the tamoxifen-sensitive breast cancer cell line MCF-7 resulted in acquired drug resistance [38].

### LncRNA UCA1 and regulation

LncRNAs are transcriptionally regulated similar to mRNAs [39, 40]. LncRNA UCA1 expression is regulated by transcription factors, Ets-2 [41], C/EBPα [42], HIF-1α [43], SATB1 [44] and transcriptional complexes, TAZ/YAP/TEAD/SMAD2/3 [45], CAPER/TBX3 [46]. Transcription factors Ets-2 [41] and C/EBPα [42] upregulate lncRNA expression in bladder cancer cells by directly binding to the core promoters and enhancing UCA1 promoter activity (Table 1). Transcription factor HIF-1α enhances lncRNA UCA1 overexpression in bladder and ovarian cancer cells in a hypoxia-dependent manner by directly binding to the hypoxia response elements (HREs) of UCA1 promoter [43]. The transcriptional complex is composed of Hippo pathway effectors (TAZ/YAP/TEAD) and members of the transforming growth factor-β (TGF-β) pathway (SMAD2/3) that are recruited to the UCA1 promoter and synergistically induce its expression in breast cancer cells after TGF-β treatment [45, 47]. Conversely, transcription factor SATB1 represses lncRNA UCA1 expression in breast cancer cells by directly binding to the promoter and 3.0 kb upstream regions of UCA1 and closing the chromatin structure, thereby blocking transcription [44]. The transcriptional complex composed of coactivator of activating protein-1 and estrogen receptors (CAPERα)/T-box 3 (TBX3) inhibits lncRNA UCA1 by binding to its promoter in cultured primary human foreskin fibroblasts (HFFs) [46].

**Table 1:** Regulators of lncRNA UCA1 expression

Regulators	Roles on lncRNA UCA1	regulation level	References
Ets-2	upregulate expression	transcriptional	Wu w, *et al*. [41]
C/EBPα	upregulate expression	transcriptional	Xue M, *et al*. [42]
TAZ/YAP/TEAD	upregulate expression	transcriptional	Hiemer SE, *et al*. [45]
HIF-1α	upregulate expression	transcriptional	Xue M, *et al*. [43]
SATB1	downregulate expression	transcriptional	Lee JJ, *et al*. [44]
CAPERα/TBX3	downregulate expression	transcriptional	Kumar PP, *et al*. [46]
miR-1	downregulate expression	post-transcriptional	Wang T, *et al*. [48]

The expression of lncRNA UCA1 is also regulated at post-transcriptional level. A recent study reported that knockdown of miR-1 resulted in Ago2-slicer-dependent lncRNA UCA1 overexpression, whereas miR-1 overexpression decreased UCA1 levels in bladder cancer cells [48]. Wang *et al*. postulated that miR-1 targets and cleaves lncRNA UCA1 in an Ago2-dependent manner similar to degradation of protein-coding mRNAs by miRNAs [48]. However, this hypothesis needs to be experimentally proven.

### LncRNA UCA1 and prognosis

The prognostic value of lncRNA UCA1 in various human malignant tumors has been evaluated by many studies. Liu *et al*. conducted a meta-analysis of 1,240 patients from 15 articles on colorectal cancer, esophageal squamous cell carcinoma, prostate cancer, hepatocellular carcinoma, non-small cell lung cancer, gastric cancer, ovarian cancer and concluded that lncRNA UCA1 was an independent prognostic factor for OS in cancer patients [49]. The meta-analysis showed that higher expression of lncRNA UCA1 (HR = 1.71, 95% CI: 1.43–1.99) was associated with poor OS based on cancer type, cut-off value, analysis type and sample size. Furthermore, the pooled odds ratios (ORs) showed that elevated lncRNA UCA1 was associated with lymph node metastasis (OR = 2.98, 95% CI: 2.06–4.30), distant metastasis (OR = 3.14, 95% CI: 1.77–5.58) and poor clinical stage (OR = 2.76, 95% CI: 2.08–3.68) [49]. However, larger-size, multi-center and higher-quality studies are necessary to validate the results in this study. Higher lncRNA UCA1 expression was also reported as an independent prognostic biomarker for pancreatic cancer [32] and glioma [33]. The poor prognosis in cancer therapy is mainly due to metastasis and drug resistance.

### Direct binding targets of LncRNA UCA1

The molecular mechanisms for most lncRNAs remain to be fully elucidated. They directly bind to DNA, RNA, or proteins, and many techniques have been developed to identify each of these interactions. LncRNA-DNA interactions can be identified by Capture Hybridization Analysis of RNA Targets (CHART) [50] and Chromatin Isolation by RNA Purification (ChIRP) [51] that involve hybridization of complementary oligonucleotides to the lncRNA of interest and serve as an affinity handle to recruit bound DNA. Similarly, radioimmunoassay sequencing (RIA-seq) is used to assay lncRNA-RNA interactions [52]. Techniques such as RNA immunoprecipitation (RIP) or its variants, cross-linking immunoprecipitation (CLIP) [53, 54], and photoactivatable ribonucleoside-enhanced cross-linking immunoprecipitation (PAR-CLIP) [55] utilize antibodies to reveal lncRNA-protein interactions. Furthermore, high-throughput methods such as *in vivo* click selective 2′ hydroxyl acylation and profiling experiment (icSHAPE) [56] or selective 2′-hydroxyl acylation analyzed by primer extension sequencing (SHAPE-seq) [57], have been developed in recent years to explore RNA secondary structure.

LncRNA UCA1 has also been reported to bind to several miRNAs in different cancer cells. These include miR-216b in hepatocellular cancer [22], miR-193a in non-small cell lung cancer [58], miR-145 in bladder cancer [59], miR-204 in colorectal cancer [34] and esophageal cancer [60], miR-1 in bladder cancer [48], miR-18a in breast cancer cells [61], miR-16 in CML [62] (Table 2).

**Table 2:** Direct binding targets of lncRNA UCA1

Direct binding targets	Tumors	Drug resistance	References	Published journals and years
miR-216b	HCC	N/A	Wang F, *et al*. [22]	*Oncotarget.2015*
miR-193a	NSCLC	N/A	Nie W, *et al*. [58]	*Cancer Lett.2016*
miR-145	bladder cancer	N/A	Xue M, *et al*. [59]	*Cancer Sci.2015*
miR-204	colorectal cancer	YES	Bian Z, *et al*. [34]	*Sci Rep.2016*
miR-204	esophageal cancer	N/A	Jiao C, *et al*. [60]	*Oncol Rep.2016*
miR-18a	breast cancer	YES	Li X, *et al*. [61]	*Tumour Biol.2016*
miR-16	CML	YES	Xiao Y, *et al*. [62]	*DNA AND CELL BIOLOGY.2016*
miR-1	bladder cancer	N/A	Wang T, *et al*. [48]	*Tumour Biol.2014*

LncRNAs play the role of a signal, decoy, guide or scaffold by binding to DNAs, RNAs and proteins [63]. These mechanisms are not mutually exclusive and lncRNAs may play one or more of the roles. For example, lncRNA UCA1 plays the role of a decoy by acting as a sponge and sink for miRNAs, which results in promoting carcinogenesis or drug resistance.

### LncRNA UCA1 in cancer drug resistance

Systemic or local chemotherapy is widely used in cancer therapy. However, intrinsic and acquired drug resistance reduces the efficacy of cancer therapies. Although remarkable progress has been made in understanding the mechanisms underlying anti-cancer drug resistance, many questions still remain unanswered. The cancer cells employ different mechanisms to effectively escape chemotherapy [64]. These include aberrant expression of glutathione transferase and topoisomerase II, reduced uptake of water-soluble drugs, enhanced DNA damage repair, enhanced drug metabolism, decreased apoptosis, and increased energy-dependent efflux of chemotherapeutic drugs, all of which reduce the effects of the cancer therapeutics [65]. In recent years, the role of lncRNAs in non-mutational regulation of gene function has been described [66]. LncRNAs bind to various writers, erasers, readers of histone modifications, and other chromatin regulatory factors [67]. LncRNA UCA1 is a good example of the role of lncRNAs in drug resistance.

### LncRNA UCA1 and bladder cancer drug resistance

Bladder cancer is ranked fourth among males and tenth among females in regard to leading causes of deaths worldwide [68]. Cisplatin and gemcitabine are the preferred drugs for chemotherapy after surgery for muscle-invasive bladder cancer patients and drug resistance greatly impedes long-term survival [69]. The molecular mechanisms promoting resistance to cisplatin/gemcitabine-based chemotherapy are only recently being identified.

In 2008, Wang *et al*. showed that transfection of a 1.4 kb full-length lncRNA UCA1 cDNA strongly promoted the proliferation, motility and invasiveness of the bladder TCC cell line, BLS-211 [21]. Stable UCA1 overexpression in BLS-211 resulted in drug resistance to cisplatin and mitomycin with their IC_50_ increased by 2.4 and 1.9 fold, respectively. Microarray analysis identified 16 upregulated and 26 downregulated genes. The genes were related to tumorigenesis and/or embryonic development with WNT6, CYP1A1, and AURKC among the upregulated genes, and MBD3 and SRPK1 among the downregulated genes. Fan *et al*. established a cisplatin-resistant T24-resistance bladder cancer cell line by continuous exposure of the T24 bladder cancer cell line to cisplatin (2.0 μg/ml) over 12 months [70]. The expression of lncRNA UCA1 was higher in the T24-cisplatin resistant cells than T24 cells [70]. Forced expression of lncRNA UCA1 in T24 cells decreased cisplatin-induced cell death, whereas knockdown of lncRNA UCA1 in T24-resistance cells increased cell death [70]. *In vitro* analysis revealed that lncRNA UCA1 increased Wnt6 expression and subsequent activation of the Wnt signaling pathway, consistent with previous findings [70]. Higher lncRNA UCA1 levels were observed in blood samples of advanced bladder cancer patients after cisplatin-based chemotherapy with a positive correlation between UCA1 and Wnt6 mRNA expression levels in the bladder cancer tissues [70]. Recently, Pan *et al*. demonstrated that forced expression of lncRNA UCA1 in the bladder cancer cell line UMUC-2 reduced apoptosis upon cisplatin/gemcitabine treatment, whereas silencing of lncRNA UCA1 in the bladder cancer cell line 5637 increased cellular apoptosis [71]. Both *in vitro* and *in vivo* experiments confirmed that lncRNA UCA1 induced cisplatin/gemcitabine resistance through activation of CREB by p-AKT and subsequent upregulation of miR-196a-5p [71]. Moreover, expression of lncRNA UCA1, miR-196a-5p and p-CREB was positively correlated in bladder cancer tissues [71].

### LncRNA UCA1 and ovarian cancer drug resistance

Ovarian cancer is ranked first in developed countries and second in developing countries as the cause of gynecological cancer-related deaths worldwide [72]. Although platinum-based chemotherapy is effective in ovarian cancer treatment, primary or secondary resistance to cisplatin severely affects therapeutic efficacy [73]. The overall 5-year survival rate of ovarian cancer is less than 50% [72]. Hence, there is a great need for discovering predictive biomarkers for ovarian cancer.

In 2015, Wang *et al*. found that lncRNA UCA1 and SRPK1 mRNA was substantially upregulated in ovarian cancer tissues compared to normal ovarian tissues [24]. Stable transfection of pcDNA-UCA1 in SKOV3 cells contributes to cisplatin resistance with a 2.41 fold increase in IC_50_, increased SRPK1 and Bcl-2 and decreased Bax, Caspase-3 and Caspase-9 expression. However, knockdown of SRPK1 overcomes cisplatin resistance in SKOV3-pcDNA-UCA1 cells and increased Bcl-2 and decreased Bax, Caspase-3 and Caspase-9 expression is observed. These results revealed that SRPK1 and apoptosis related proteins played an important role in the lncRNA UCA1 induced cisplatin resistance [24]. Furthermore, Zhang *et al*. observed that lncRNA UCA1 levels were significantly higher in ovarian cancer tissues compared to normal ovarian tissues, and high UCA1 expression was associated with more lymph node metastasis, advanced FIGO stage, and bad response to platinum-based chemotherapy [74].

### LncRNA UCA1 and breast cancer drug resistance

Breast cancer is the most commonly diagnosed malignant tumor among women worldwide with nearly 70% of it being estrogen receptor positive [75]. Tamoxifen is one of the major hormone therapies for ER positive breast cancer in clinical practice. However, tamoxifen resistance limits the long-term effects of the treatment of ER positive breast cancer. Hence, it is necessary to identify the molecular mechanisms underlying tamoxifen resistance.

Wu *et al*. showed that knockdown of lncRNA UCA1 in tamoxifen resistant LCC2 and LCC9 cells increased apoptosis upon tamoxifen treatment accompanied by significant reduction in p-AKT and p-mTOR [76]. However, forced expression of lncRNA UCA1 in tamoxifen-sensitive MCF-7 cells substantially reduced tamoxifen induced apoptosis, which could be abrogated by the mTOR specific inhibitor rapamycin [76]. These results revealed that lncRNA UCA1 induced tamoxifen resistance in breast cancer cells partly through activation of the mTOR signal pathway [76]. Further, Li *et al*. showed that ectopic expression of lncRNA UCA1 in MCF-7 induced acquired tamoxifen resistance [61]. In addition, functional analysis demonstrated that lncRNA UCA1 acted as a sponge for miR-18a, which is an important modulator of tamoxifen resistance by regulating cell cycle proteins. Interestingly, tamoxifen treatment upregulated lncRNA UCA1 in MCF-7 cells through a miR-18a-HIF1α feedback loop [61]. Moreover, Liu *et al*. showed that lncRNA UCA1 expression was upregulated in the tamoxifen resistant breast cancer cells compared to their parental MCF-7 and T47D cells [77]. Stable transfection of lncRNA UCA1 in MCF-7 and T47D cells resulted in drug resistance to tamoxifen, whereas siRNA targeting lncRNA UCA1 enhanced tamoxifen sensitivity. Also, knockdown of lncRNA UCA1 prevented the nuclear translocation of β-catenin, thereby inhibiting the Wnt signaling pathway [77]. This suggested that lncRNA UCA1 induced tamoxifen resistance by increasing the activity of Wnt/β-Catenin signaling [77]. Further, Xu *et al*. demonstrated that treatment of MCF-7 cells with exosomes released from tamoxifen resistant LCC2 cells with lncRNA UCA1 resulted in enhanced cell viability accompanied by decreased expression of cleaved caspase-3 after tamoxifen treatment [38]. Further, LCC2 exosomes with impaired UCA1 could not induce tamoxifen resistance in MCF-7 cells. These results showed that exosome-mediated transfer of lncRNA UCA1 induced acquired tamoxifen resistance in MCF-7 cells [38].

### LncRNA UCA1 and lung cancer drug resistance

Currently, EGFR-TKIs like gefitinib or erlotinib are the first-line treatments against advanced non-small cell lung cancer (NSCLC) harboring EGFR-activating mutations [78, 79, 80]. However, the majority of EGFR-mutated NSCLCs that respond to initial EGFR-TKI therapy develop acquired resistance in about 12 months [81, 82]. Functional analysis indicated that a secondary T790M mutation is the major cause of acquired resistance ; c-MET amplification, PIK3CA mutations (∼5%), BRAF mutations and small-cell lung cancer transformation were also associated with acquired resistance [83–85].

Cheng *et al*. performed microarray expression profiling of lncRNAs/mRNA and demonstrated high lncRNA UCA1 expression in acquired gefitinib-resistant PC9/R cells compared to the parental gefitibib-sensitive PC9 cells [86]. Further, Cheng *et al*. demonstrated higher lncRNA UCA1 expression in PC9/R and H1975 cells with acquired resistance compared to A549, H460, H23 and H1299 cells with primary resistance [87]. The lncRNA UCA1 levels were higher in lung cancer tissues from patients with acquired resistance to EGFR-TKIs compared to EGFR-TKI sensitive patients or patients with primary resistance to EGFR-TKIs [87]. Functional analysis showed that knockdown of UCA1 in PC9/R cells without T790M mutation partly restored gefitinib sensitivity with increased expression of caspase 3 and caspase 8, whereas H1975 cells with T790M mutation remained gefitinib resistant. Moreover, both *in vitro* and *in vivo* analysis confirmed that lncRNA UCA1 contributed to non-T790M acquired resistance to EGFR-TKIs by activating the AKT/mTOR pathway and EMT [87].

### LncRNA UCA1 and gastric cancer drug resistance

Gastric cancer is the most frequently diagnosed cancer of the digestive system with high morbidity and mortality [88]. For advanced gastric cancer, both pre-operative and post-operative chemotherapies are critical for survival [89]. However, the underlying mechanisms are unclear.

In 2016, Fang *et al*. showed increased lncRNA UCA1 and decreased miR-27b in MDR gastric cancer cells such as SGC-7901/ADR, SGC-7901/DDP, and SGC- 7901/5-FU cells compared to their parental SGC-7901 cells, whereas knockdown of lncRNA UCA1 substantially restored miR-27b expression [90]. Bioinformatic analysis demonstrated two possible binding sites of mirR-27b in lncRNA UCA1 and *in vivo* analysis of gastric cancer tissues confirmed the negative correlation between lncRNA UCA1 and miR-27b expression [90]. Furthermore, lncRNA UCA1 silencing and miR-27b overexpression overcame the drug resistance by reducing the IC_50_ of ADR, DDP, and 5-FU in SGC-7901/ADR cells, which was accompanied with enhanced apoptosis due to decreased Bcl-2 and increased cleaved caspase-3 levels [90]. In addition, Shang *et al*. showed that lncRNA UCA1 levels were higher in SGC7901/ADR cells compared to the parental SGC7901 cells and this resulted in a 4.3 fold increase in IC_50_ after adriamycin treatment [91]. However, knockdown of lncRNA UCA1 suppressed this resistance to adriamycin in SGC7901/ADR and increased cellular apoptosis upon adriamycin treatment due to increased cleaved PARP and decreased Bcl-2 [91].

### LncRNA UCA1 and colorectal cancer drug resistance

Colorectal cancer is the third most common cancer in males and the second most common cancer in females worldwide [72]. The initiation and progression of CRC is a multi-step process controlled by the deregulation of multiple oncogenes and tumor suppressors [92]. However, diagnostic methods and therapies are needed to improve CRC detection and survival.

Bian *et al*. showed that ectopic expression of lncRNA UCA1 in SW480 cells resulted in chemoresistance with reduction in 5-Fu induced apoptosis, whereas knockdown of lncRNA UCA1 in HCT116 cells enhanced apoptosis [34]. Bioinformatic analysis showed that lncRNA UCA1 has a miR-204-5p recognition sequence. Previous *in vitro* results showed that miR-204-5p enhanced chemotherapeutic sensitivity of CRC cells. Luciferase and RIP assays demonstrated direct binding between UCA1 and miR-204-5p [34]. Mechanistically, lncRNA UCA1 acted as a sponge of miR-204-5p and upregulated the expression of miR-204-5p target genes (BCL2, RAB22A and CREB1), thereby contributing to 5-Fu resistance. Moreover, positive correlation between CREB1 and UCA1 and inverse correlation between CREB1 and miR-204-5p was observed in CRC tissues, thereby suggesting that UCA1 acted as a ceRNA of miR-204-5p to regulate CREB1 expression in CRC tumors [34].

### LncRNA UCA1 and prostate cancer drug resistance

Prostate cancer is the second most common cancer in males in the world with good prognosis [72]. However, the prognosis of some advanced or metastatic patients is poor and drug resistance is one of the major reasons.

Wang *et al*. showed that lncRNA UCA1 overexpression downregulated miR-204 expression in LNCaP cells and 22RV1 cells, while miR-204 upregulation substantially decreased Sirt1 expression suggesting that miR-204 inversely modulated Sirt1 expression [30]. In addition, lncRNA UCA1 overexpression increased Sirt1 expression in PNT2 cells, while silencing of endogenous lncRNA UCA1 decreased Sirt1 expression in LNCaP and 22RV1 cells [30]. This suggested a UCA1/miR-204/Sirt1 axis in LNCaP and 22RV1 cells. Interestingly, lncRNA UCA1 and Sirt1 levels were significantly upregulated in 22RV1/DR cells compared to the parental 22RV1 cells and miR-204 was downregulated. Transfection with UCA1 and Sirt1 siRNAs or miR-204 mimics in 22RV1/DR cells significantly decreased the expression of P-glycoprotein (P-gp), which is an important membrane pump responsible for drug resistance [30]. Additionally, reduced docetaxel IC_50_ in 22RV1/DR cells due to increased cleaved caspase-3 expression and enhanced apoptosis was observed [30]. These results revealed that lncRNA UCA1 induced docetaxel resistance in prostate cancer cells via the UCA1/miR-204/Sirt1 axis.

### LncRNA UCA1 and CML drug resistance

To date, imatinib has been a revolutionary treatment against chronic myelogenous leukemia (CML) with about 95% of CML patients achieving remission after imatinib therapy [93, 94]. However, some patients in accelerated phase or blast crisis demonstrate drug resistance to imatinib [94, 95]. The underlying mechanisms of imatinib resistance could be a result of point mutations in the breakpoint cluster region-Abelson murine leukemia (BCR-ABL) kinase domain, amplification of the BCR-ABL gene, and overexpression of the multidrug resistance protein 1 (MRP1) gene in tumor cells [96, 97].

In 2017, Xiao *et al*. showed that K562/IM cells displayed a 10 fold increase in IC_50_ compared to a 5 fold increase in IM resistance of K562/IMR cells in relation to the parent K562 cells whereas [62]. Further, MDR1 protein expression was higher in K562/IM-R and lower in K562/IM cells compared to K562 cells. Also, lncRNA UCA1 expression was higher in K562/IM-R and lower in K562/IM cells than in K562 cells, which indicated the role of lncRNA UCA1 in IM resistance of CML cells [63]. Stable transfection of lncRNA UCA1 in K562 cells markedly upregulated MDR1 mRNA and protein levels and resulted in IM resistance, whereas silencing of UCA1 in K562/IM cells significantly inhibited MDR1 expression. Thus, lncRNA UCA1 emerged as a competitive endogenous RNA (ceRNA) of MDR1 that induced IM resistance by sequestering miR-16 [62].

**Figure 1:**
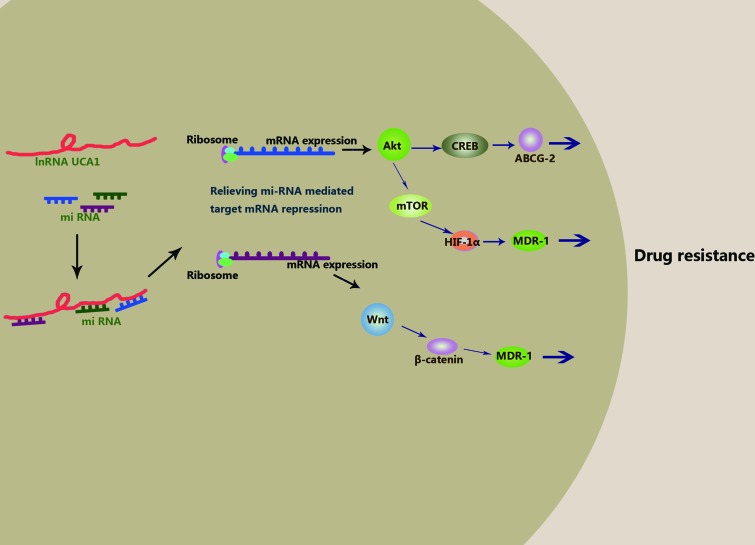
Hypothesis of molecular mechanism of lncRNA UCA1 in anti-cancer drug resistance

Taken together, these studies confirm the pivotal role of lncRNA UCA1 in anti-cancer drug resistance to cisplatin, gemcitabine, 5-FU, tamoxifen, imatinib and EGFR-TKIs. However, the underlying molecular mechanisms still remain to be investigated in greater detail (Table 3). Therefore, based on current knowledge, we postulate that lncRNA UCA1 promotes drug resistance by directly binding to specific miRNAs and acting as a “sponge”. To date, miR-216b, miR-193a, miR-145, miR-204, miR-1, miR-18a and miR-16 (Table 2) have been shown to directly bind to lncRNAs. Among these, miR-204, miR-18a and miR-16 have been verified to bind to lncRNA UCA1 directly and then induce drug resistance. In future, computer prediction, RIA-seq screening and luciferase and RIP assays should be performed to comprehensively identify specific miRNAs that bind to lncRNA UCA1 specifically. Next, the interactions between lncRNA UCA1 and miRNAs could relieve miRNAs mediated target mRNAs repression. Up to now, there is none concrete target mRNAs reported in these literatures. Maybe, SRPK1, AKT, Wnt6 belong to this kind of target mRNAs. And then, the overexpression of the target proteins results in activation of signaling pathways that decrease apoptosis, enhance proliferation or reduce cell cycle arrest. Based on available data, several signaling pathways like AKT/mTOR/HIF-1a, AKT/CREB and Wnt/β-catenin could be involved in the process (Figure 1). It is a systematic and thorough work to elucidate this network in great need of basic and clinical research.

**Table 3:** LncRNA UCA1 in anti-cancer drug resistance

Cancer types	Drugs	Direct targets	Mechanisms	References	Published Journals and years
bladder cancer	cisplatin/mitomycin	N/A	N/A	Wang F, *et al*. [21]	*FEBS Lett.2008*
bladder cancer	cisplatin	N/A	Wnt6/Wnt pathway	Fan Y, *et al*. [70]	*FEBS J.2014*
bladder cancer	cisplatin/gemcitabine	N/A	p-AKT/CREB/miR-196a-5p/p27Kip1	Pan J, *et al*. [71]	*Cancer Lett.2016*
breast cancer	tamoxifen	N/A	N/A	Xu CG, *et al*. [38]	*Eur Rev Med Pharmacol Sci.2016*
breast cancer	tamoxifen	N/A	mTOR pathway	Wu C, *et al*. [76]	*Med Sci Monit.2016*
breast cancer	tamoxifen	miR-18a	miR-18a, HIF1α	Li X, *et al*. [61]	*Tumour Biol.2016*
breast cancer	tamoxifen	N/A	Wnt/β-Catenin	Liu H, *et al*. [77]	*PLoS One.2016*
CML	imatinib	miR-16	miR-16/MDR1	Xiao Y, *et al*. [62]	*DNA Cell Biol.2016*
colorectal cancer	5-FU	miR-204-5p	miR-204-5p/CREB1,Bcl2,RAB22A	Bian Z, *et al*. [34]	*Sci Rep.2016*
gastric cancer	doxorubicin/cisplatin/5-FU	N/A	miR-27b/Bcl-2,caspase-3	Fang Q, *et al*. [90]	*Med Sci Monit.2016*
gastric cancer	adriamycin	N/A	Bcl-2,PARP	Shang C,*et al*. [91]	*Cancer Chemother Pharmacol.2016*
lung cancer	EGFR-TKIs	N/A	AKT/mTOR/caspase-3,caspase-8	Cheng N,*et al*. [87]	*Oncotarget.2015*
ovarian cancer	cisplatin	N/A	SRPK1/Bcl-2,Bax,caspase-3,caspase-9	Wang F, *et al*. [24]	*Neoplasma.2015*
ovarian cancer	platinum-based chemotherapy	N/A	N/A	Zhang L,*et al*. [74]	*Cancer Chemother Pharmacol.2016*
prostate cancer	docetaxel	N/A	miR-204/Sirt1	Wang X,*et al*. [30]	*Cancer Chemother Pharmacol.2016*

### Future clinical applications

#### LncRNA UCA1 as a cancer biomarker

LncRNAs are promising diagnostic or predictive biomarkers for human cancers because of their specific expression and critical role in cancer initiation and progression. LncRNA UCA1 functions as an oncofetal gene similar to lncRNA H19 [98] and is detected not only in tumor tissue samples but also in blood and urine samples from bladder cancer patients as a circulating biomarker [35]. After birth, lncRNA UCA1 is undetectable in majority of normal tissues [19, 21]. LncRNA UCA1 is reactivated in various malignant tumors including bladder cancer, breast cancer, cervical cancer, colorectal cancer, esophageal squamous cell carcinoma, hepatocellular carcinoma, gastric cancer, lung cancer, melanoma, ovarian cancer, thyroid cancer, tongue squamous cell carcinoma, prostate cancer, acute myeloid leukemia, glioma, pancreatic cancer and osteosarcoma [19–33]. Hence, lncRNA UCA1 in body fluid samples is a potential diagnostic and prognostic biomarker for tumor initiation, metastasis and recurrence of cancers. RT-PCR based lncRNA UCA1 expression detection in urine samples is an effective and noninvasive assay with high sensitivity (100%) and specificity (67%) for the diagnosis of bladder cancer with the standard cytology method [36]. However, the diagnostic value of lncRNA UCA1 in body fluid samples for other types of malignant tumors remains to be confirmed.

The positive correlation between lncRNA UCA1 expression and poor prognosis in a great number of cancer types as mentioned in the above meta-analysis suggests that lncRNA UCA1 could act as an independent prognostic factor for cancer patients [49]. However, larger-size, multi-center and higher-quality studies with a unified criterion for determining lncRNA UCA1 expression are necessary to validate and confirm these results for the different kinds of cancers.

Further, many studies show that lncRNA UCA1 induces drug resistance in various cancers such as bladder cancer, ovarian cancer, breast cancer, lung cancer, gastric cancer, colorectal cancer, prostate cancer and CML [21, 24, 30, 34, 38, 61, 62, 70, 71, 74, 76, 77, 87, 90, 91], thereby greatly reducing the efficacy of cancer therapy. The majority of these results of drug resistance were from *in vitro* studies, whereas clinical data was available only in ovarian cancers where high lncRNA UCA1 expression was associated with the response to platinum-based chemotherapy [74].

Taken together, lncRNA UCA1 is potentially a good broad spectrum biomarker for cancer diagnosis, prognosis or therapeutic monitoring. However, none of the lncRNA UCA1 products are in clinical trials yet. Therefore, we propose that further animal model studies and prospective clinical studies will highlight and describe the role of lncRNA UCA1 in anti-cancer drug resistance leading to its investigation for various clinical applications in the future.

### LncRNA UCA1 related therapies against cancers

Although the role of lncRNAs in cancer drug resistance is only beginning to be discovered, the application potential of lncRNA UCA1 as a candidate to develop novel strategies to reverse drug sensitivity to chemotherapy or molecular targeted therapy cannot be ignored.

### LncRNA UCA1 targeted therapeutics (RNAi and ASO)

RNA targeted therapeutics include natural or artificially synthesized oligonucleotides that target mRNA for therapy or elucidate gene functions [99]. Currently, small molecules and antibodies have been used to target proteins, while oligonucleotides target various RNAs [99]. The two main approaches in RNA targeted therapeutics include double stranded RNA-mediated interference (RNAi) and antisense oligonucleotides (ASO). RNAi induces lncRNA degradation, while ASO sterically impedes RNA splicing, formation of functional protein complexes and the formation or function of a mature RNA [100]. Both RNAi and ASO have been verified in clinical trials for many diseases such as cancer and neurodegeneration [99]. Most studies have reported that lncRNA UCA1 is also specifically expressed in various cancer cells and involved in drug resistance [21, 24, 30, 34, 38, 61, 62, 70, 71, 74, 76, 77, 87, 90, 91]. Hence, we postulate that targeting lncRNA UCA1 may partially or completely restore drug sensitivity and improve the efficacy of chemotherapy. In fact, knockdown of lncRNA UCA1 by short interfering RNAs (siRNA) or short hairpin RNA (shRNA) has been shown to reverse drug resistance in various cancer cells such as bladder cancer [70, 71], breast cancer [38, 76, 77], lung cancer [87], gastric cancer [90, 91], colorectal cancer [34], prostate cancer [30], CML [62], ovarian cancer [24]. Also, miR-1 has been shown to suppress lncRNA UCA1 expression in bladder cancer cells [48].

### Transcription factor or transcription complex targeted therapeutics

A number of studies have demonstrated that lncRNA UCA1 expression is suppressed by the transcription factor SATB1 [44] and transcriptional complex composed of coactivator of activating protein-1 and estrogen receptors (CAPERα) and T-box 3 (TBX3) [46]. Hence, SATB1 and CAPERα/TBX3 can be potentially exploited to inhibit the expression of lncRNA UCA1 and reverse drug resistance. Apart from these, many studies have indicated that the expression of lncRNA UCA1 is upregulated by transcription factors Ets-2 [41], C/EBPα [42], HIF-1α [43], and transcriptional complexes (TAZ/YAP/TEAD/SMAD2/3) [45]. Thus, Ets-2, C/EBPα, HIF-1α, and transcriptional complexes (TAZ/YAP/TEAD/SMAD2/3) are potential targets to knockdown the expression of lncRNA UCA1.

### Signaling pathway and effector molecule targeted therapeutics

Many studies in the last two decades have explored the resistance mechanisms in cancer cells and approaches to reverse drug resistance. These studies have identified the role of proteins such as MDR1, ABCG2, and MRP in cancer drug resistance, and some have been exploited to develop various strategies for cancer therapy. Recently, the role of non-coding RNAs such as microRNAs and lncRNAs has been confirmed in drug resistance [101, 102]. The miRNAs such as miR-204, miR-18a, miR-27b and miR-16 have demonstrated the therapeutic potential to restore drug sensitivity, whereas miRNAs such as miR-196a and proteins such as mTOR, MDR1, P27, BCL2, SRPK1, Sirt1, β-Catenin and Wnt are targets for cancer therapy. Preliminary studies have shown the therapeutic potential of lncRNA UCA1 in cancer therapy, especially drug resistance.

### CRISPR/Cas9-based therapeutics

Direct targeting of the UCA1 genomic locus is another method to knockdown UCA1 expression in malignant tumors. Tsui-Ting *et al*. silenced lncRNA UCA1 expression in HCT-116 human colon cancer cells by using the (CRISPR)/CRISPR-associated (Cas) genome editing system, which involves clustered regularly interspaced short palindromic repeats [103]. Moreover, Zhen *et al*. designed gRNAs specific to UCA1 and demonstrated significant knockdown of lncRNA UCA1 when transfected into 5637 and T24 bladder cancer cells with CRISPR/Cas9 systems targeting UCA1 [104]. Hence, CRISPR/Cas9 systems are potential tools to inhibit the expression of lncRNA UCA1 at genomic level to attenuate drug resistance in research.

Taken together, potential novel strategies to reverse drug sensitivity to chemotherapy or molecular targeted therapy include lncRNA UCA1 targeted therapeutics, CRISPR/Cas9 system, transcription factor or transcription complex targeted therapeutics, signaling pathway and effector molecule targeted therapeutics.

## CONCLUSIONS

LncRNA UCA1 expression is associated with a number of anti-cancer drug resistance tumors, however, the mechanisms remain to be elucidated in greater detail, especially from a clinical standpoint. Yet, lncRNA UCA1 shows great potential as a diagnostic, predictive or prognostic biomarker, and a therapeutic target in malignant tumors.

## Abbreviations

lncRNA: long non coding RNA
UCA1: urothelial carcinoma associated 1
UCA1a: urothelial carcinoma associated 1a
ORFs: open reading frames
CUDR: cancer upregulated drug resistant gene
TAZ: Transcriptional coactivator with PDZ-binding motif
YAP: yes-associated protein
TEAD: TEA domain family member
ceRNA: competing endogenous RNA
CAPER/TBX3: coactivator of activating protein-1 and estrogen receptors
C/EBPα: CCAAT/enhancer-binding protein α
HIF-1α: hypoxia-inducible factor 1 α
HREs: hypoxia response elements
TGF-β: transforming growth factor-β
HFFs: human foreskin fibroblasts
RIA-seq: radioimmunoassay sequencing
CHART: capture hybridization analysis of RNA targets
ChIRP: chromatin isolation by RNA purification
RIP: RNA immunoprecipitation
CLIP: cross-linking immunoprecipitation
PAR-CLIP: photoactivatable ribonucleoside–enhanced cross-linking immunoprecipitation
icSHAPE: *in vivo* click selective 2′ hydroxyl acylation and profiling experiment
SHAPE-seq: selective 2′-hydroxyl acylation analyzed comby primer extension sequencing
BCR-ABL: breakpoint cluster region-Abelson murine leukemia
CML: Chronic Myeloid Leukemia
NSCLC: non-small cell lung cancer
5-Fu: 5-fluorouracil
ABCB1: ATP binding cassette subfamily B member 1
MRP1: multi-drug resistant associate protein 1
MDR1: multi-drug resistant protein
N/A: not available.
